# Increasing phylogenetic resolution at low taxonomic levels using massively parallel sequencing of chloroplast genomes

**DOI:** 10.1186/1741-7007-7-84

**Published:** 2009-12-02

**Authors:** Matthew Parks, Richard Cronn, Aaron Liston

**Affiliations:** 1 Department of Botany and Plant Pathology, Oregon State University, Corvallis, OR, 97331, USA; 2 Pacific Northwest Research Station, USDA Forest Service, Corvallis, OR, 97331, USA

## Abstract

**Background:**

Molecular evolutionary studies share the common goal of elucidating historical relationships, and the common challenge of adequately sampling taxa and characters. Particularly at low taxonomic levels, recent divergence, rapid radiations, and conservative genome evolution yield limited sequence variation, and dense taxon sampling is often desirable. Recent advances in massively parallel sequencing make it possible to rapidly obtain large amounts of sequence data, and multiplexing makes extensive sampling of megabase sequences feasible. Is it possible to efficiently apply massively parallel sequencing to increase phylogenetic resolution at low taxonomic levels?

**Results:**

We reconstruct the infrageneric phylogeny of *Pinus *from 37 nearly-complete chloroplast genomes (average 109 kilobases each of an approximately 120 kilobase genome) generated using multiplexed massively parallel sequencing. 30/33 ingroup nodes resolved with ≥ 95% bootstrap support; this is a substantial improvement relative to prior studies, and shows massively parallel sequencing-based strategies can produce sufficient high quality sequence to reach support levels originally proposed for the phylogenetic bootstrap. Resampling simulations show that at least the entire plastome is necessary to fully resolve *Pinus*, particularly in rapidly radiating clades. Meta-analysis of 99 published infrageneric phylogenies shows that whole plastome analysis should provide similar gains across a range of plant genera. A disproportionate amount of phylogenetic information resides in two loci (*ycf*1, *ycf*2), highlighting their unusual evolutionary properties.

**Conclusion:**

Plastome sequencing is now an efficient option for increasing phylogenetic resolution at lower taxonomic levels in plant phylogenetic and population genetic analyses. With continuing improvements in sequencing capacity, the strategies herein should revolutionize efforts requiring dense taxon and character sampling, such as phylogeographic analyses and species-level DNA barcoding.

## Background

Molecular phylogenetic and phylogeographic analyses are typically limited by DNA sequencing costs, and this forces investigators to choose between dense taxon sampling with a small number of maximally informative loci, or genome-scale sampling across a sparse taxon sample [1-4]. Balancing these choices is particularly difficult in studies focused on recently diverged taxa or ancient rapid radiations, as taxon sampling needs to be sufficiently large to define the magnitude of intraspecific variation and the phylogenetic depth of shared alleles [5,6]. Similarly, broad genome sampling is necessary to offset the low level of genetic divergence among individuals of recent co-ancestry and to overcome low phylogenetic signal to noise ratios characteristic of rapid radiations [6]. Next generation DNA sequencing is poised to bring the benefits of affordable genome-scale data collection to such studies at low taxonomic levels (genera, species, and populations). Massively parallel sequencing (MPS) has increased per instrument sequence output several orders of magnitude relative to Sanger sequencing, with a proportional reduction in per-nucleotide sequencing costs [7,8]. In principle this could allow the rapid sequencing of large numbers of entire organellar genomes (chloroplast or mitochondria) or nuclear loci, and result in greatly increased phylogenetic resolution [9]. To date, comparatively few plant or animal evolutionary genetic analyses have utilized MPS [10-12], due to associated costs and the technical challenge of assembling large contiguous sequences from micro-reads. These barriers have been largely eliminated through four innovations: development of strategies for targeted isolation of large genomic regions [9,13-15]; harnessing the capacity of these platforms to sequence targeted regions in multiplex [9,14,16]; streamlining sample preparation and improving throughput [17]; and developing accurate *de novo *assemblers that reduce reliance upon a predefined reference sequence [18,19].

In this paper we demonstrate the feasibility and effectiveness of MPS-based chloroplast phylogenomics for one-third of the world's pine species (*Pinus*), a lineage with numerous unresolved relationships based on previous cpDNA-based studies [20-22]. We also highlight the broad applicability of our approach to other plant taxa, and remark on the potential applications to similar mitochondrial-based studies in animals and plant DNA barcoding. Using multiplex MPS approaches, we sequenced nearly-complete chloroplast genomes (120 kilobases (kb) each total length) from 32 species in *Pinus *and four relatives in Pinaceae. Our sampling of *Pinus *includes both subgenera (subg. *Pinus*, 14 accessions; subg. *Strobus*, 21 accessions) and species exemplars chosen from all 11 taxonomic subsections [21] to evenly cover the phylogenetic diversity of the genus. Taxon density is highest for a chosen subsection (subsect. *Strobus*) as representative of a species-rich clade lacking phylogenetic resolution in previous studies [5,21-23]. Three species are also represented by two chloroplast genomes each (*P. lambertiana, P. thunbergii, P. torreyana*).

## Results

### Genomic Assemblies and Alignment

Assemblies in subgenus *Strobus *averaged 117 kb, with an estimated 8.8% missing data (compared to *P. koraiensis *reference); subg. *Pinus *assemblies averaged just less than 120 kb (6% estimated missing data, compared to *P. thunbergii *reference). Outgroup assemblies averaged just over 119 kb (10.4% average estimated missing data compared to *P. thunbergii *reference). Median coverage depth for determined positions was variable but typically high (range 21 to 156×) (Table 1, [also see additional file 1]). Full alignment of all assemblies was 132,715 bp in length, including 62,298 bp from exons encoding 71 conserved protein coding genes (20,638 amino acids), 36 tRNAs and 4 rRNAs. A high degree of co-linearity is inferred for these genomes due to the absence of major rearrangements within *de novo *contigs, and by the overall success of the polymerase chain reaction-based sequence isolation strategy (indicating conservation of the order of anchor genes containing primer sites). However, minor structural changes (a tandem duplication in two species [24] and the apparent loss of duplicate copies of *psa*M and *rps*4 in *P. koraiensis*) could not be confirmed. No evidence of interspecific recombination was detected, consistent with the rarity of recombination in plant plastomes [25].

**Table 1:** Multiplex tags and read count for sampled accession.

Accession	Multiplex Tag	Number of Reads	Read Length (bp, without tag)	Median coverage
*Abies firma*	AGCT	3110857	36	116
*Cedrus deodara*	CCCT	1338443	36	74
*Larix occidentalis*	GGT	719060	33	30
*Picea sitchensis*	ATT/AATT	1268688/710117	33/37	80
*Pinus albicaulis*	AGCT	869509	36	54
*P. aristata*	ACGT	1884108	36	100
*P. armandii*	AGCT	1233280	36	109
*P. attenuata*	ACGT	1230397	36	64
*P. ayacahuite*	CCCT	1173420	36	96
*P. banksiana*	AGCT	2307302	36	65
*P. canariensis*	CCCT	1069293	36	95
*P. cembra*	CTGT	1166707	36	40
*P. contorta*	CCT	1423631/423905	33/37	65
*P. chihuahuana*	CTGT	950336	36	21
*P. flexilis*	GGGT	1545509	36	136
*P. gerardiana*	GGT	1336725	33	98
*P. krempfii*	AAT	1569301	33	112
*P. lambertiana *N	ATT	1426598/1443555	33/37	99
*P. lambertiana *S	CCCT	1180289	36	113
*P. longaeva*	CCT	930078	33	89
*P. merkusii*	ATT	632411/585832	33/37	37
*P. monophylla*	GGT	1233556	33	145
*P. monticola*	CTGT	1460934	36	75
*P. nelsonii*	AAT	1139491/329838	33/37	81
*P. parviflora*	CCCT	920102	36	45
*P. peuce*	TACT	1402996	36	98
*P. pinaster*	GGT	1745043	33	77
*P. ponderosa*	CCT	16859450	33	44
*P. resinosa*	GGGT	2145134	36	48
*P. rzedowskii*	TACT	2419507	36	156
*P. sibirica*	CTGT	947216	36	60
*P. squamata*	TACT	1956311	36	97
*P. strobus*	GGGT	864197	36	42
*P. taeda*	CGT	1305703/1219158	33/37	90
*P. thunbergii*	AAT	1850050/2690553	33/37	104
*P. torreyana *ssp. *torreyana*	CTGT	1114111	36	76
*P. torreyana *ssp. *insularis*	ACGT	1157851	36	88

"/" indicates accession was multiplex sequenced in two sequencing runs. Median coverage is reported for determined positions (≥ 2× coverage depth) in reference-guided analysis.

**Figure 1:**
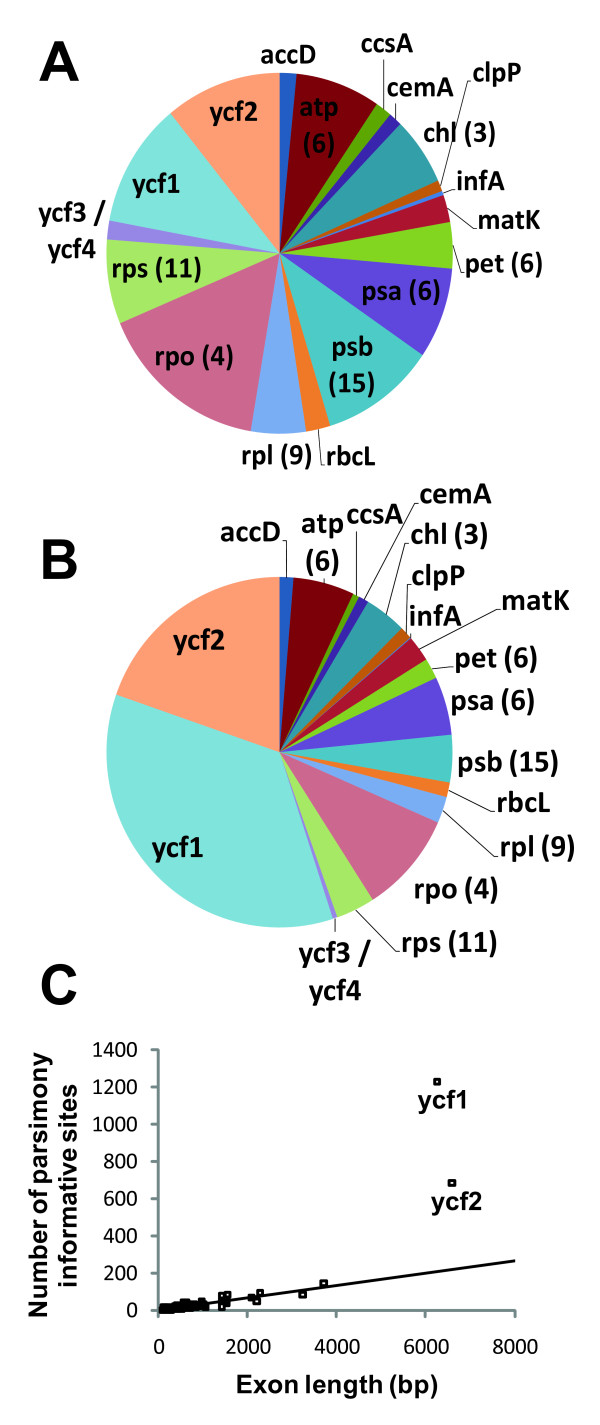
**Length and information content of 71 exons common to *Pinus *accessions sampled in this study**. **A) **Exon contributions to length as proportion of total exome length. **B) **Exon contributions to parsimony informative sites as proportion of total exome parsimony informative sites. **C) **Distribution of exons in relation to length and parsimony informative sites. In A) and B) most exons are shown by functional group (i.e., atp(), psb(); number of corresponding loci indicated in parentheses) for visualization purposes. In C) all exons were treated individually (N = 71). Trendline in C) based on all exons with exception of *ycf*1 and *ycf*2 to emphasize their departure from trend in other exons.

The aligned matrix contained 7,761 parsimony informative ingroup substitutions (4,286 non-coding positions and 3,475 coding positions) (Table 2). Over one-half of parsimony informative sites (55.0%) in protein coding regions resided in *ycf*1 and *ycf*2, two large genes of uncertain function [26], that accounted for 22% of all exon sequence (Figure 1A, B). No other exons in the pine plastome exhibit such a disproportionate number of parsimony informative sites (Figure 1C). These loci have an elevated nonsynonymous substitution rate (Table 3) and appear to have a substantial number of indels in *Pinus*, although it was not possible in many cases to confidently score indels in these loci due to the inherent limitations of reference-guided assembly of short reads in length variable regions. Start codon position, overall length and stop codon positions were nonetheless largely preserved in these loci across the genus. In addition to substitutions in exons, 48 ingroup exon indels and 23 ingroup stop codon shifts were identified in 26 loci.

**Table 2:** Summary of variable and parsimony informative sites in data partitions.

Treatment	Aligned length	Pines only Variable positions (% of total)	PI positions (% of total)	Pines and outgroups Variable positions (% of total)	PI positions (% of total)
All Nucleotides	132085	11179 (8.5)	7761 (5.9)	22834 (17.3)	11534 (8.7)
All Nucleotides without *ycf*1, *ycf*2	118935	8755 (7.4)	5852 (4.9)	18978 (16.0)	9038 (7.6)
Exon Nucleotides	62298	4716 (7.6)	3475 (5.6)	8346 (13.4)	4867 (7.8)
Exon Nucleotides without *ycf*1, *ycf*2	49044	2291 (4.7)	1566 (3.2)	4489 (9.2)	2381 (4.9)
*ycf*1	6355	1514 (23.8)	1227 (19.3)	2165 (34.1)	1507 (23.7)
*ycf*2	6794	910 (13.4)	682 (10.0)	1686 (24.8)	987 (14.5)
*ycf*1+*ycf*2	13149	2424 (18.4)	1909 (14.5)	3851 (29.3)	2494 (19.0)
Wang et al. [22]	3513	196 (5.6)	127 (3.6)	482 (13.5)	243 (6.8)
Gernandt et al. [21]	2817	197 (7.0)	128 (4.5)	345 (12.2)	167 (5.9)
Eckert and Hall [20]	3288	217 (6.6)	123 (3.7)	411 (12.5)	206 (6.3)

Data from Gernandt et al. [21] and Eckert and Hall [20] pruned to include only ingroup species and outgroup genera common to our study. (PI = parsimony informative.)

**Table 3:** Codon-based Z-test for selection results for exon sequences.

exon	*P *value H_A_: dN > dS	*P *value H_A_: dN < dS	test statistic	exon	*P *value H_A_: dN > dS	*P *value H_A_: dN < dS	test statistic
*acc*D	1	0.2013	0.8400	*psb*K	0.3925	1	0.2735
*atp*A	1	**0.0146**	2.2071	*psb*L	0.0922	1	1.3350
*atp*B	1	**0.0007**	3.2809	*psb*M	**0.0125**	1	2.2697
*atp*E	0.0632	1	1.5390	*psb*N	1	0.1632	0.9854
*atp*F	0.0888	1	1.3559	*psb*T	1	0.1193	1.1842
*atp*H	1	**0.0210**	2.0561	*psb*Z	1	0.0783	1.4253
*atp*I	1	0.0622	1.5477	*rbc*L	1	**0.0000**	4.5278
*ccs*A	1	0.1785	0.9248	*rpl*2	1	**0.0031**	2.7867
*cem*A	1	0.2453	0.6915	*rpl*14	1	**0.0234**	2.0097
*chl*B	1	**0.0002**	3.6305	*rpl*16	1	**0.0463**	1.6957
*chl*L	1	**0.0039**	2.7022	*rpl*20	1	**0.0359**	1.8161
*chl*N	1	**0.0000**	5.9654	*rpl*22	1	**0.0057**	2.5720
*clp*P	0.4634	1	0.0920	*rpl*23	1	0.2150	0.7919
*inf*A	1	0.1554	1.0177	*rpl*32	1	0.1692	0.9613
*mat*K	1	0.1628	0.9871	*rpl*33	1	0.0695	1.4893
*pet*A	1	**0.0140**	2.2233	*rpl*36	1	0.1550	1.0194
*pet*B	1	**0.0022**	2.9021	*rpo*A	1	0.0691	1.4928
*pet*D	1	0.1025	1.2742	*rpo*B	1	**0.0000**	4.2298
*pet*G	1	0.0697	1.4881	*rpo*C1	1	**0.0103**	2.3448
*pet*L	0.0791	1	1.4197	*rpo*C2	1	**0.0017**	2.9858
*pet*N	1	0.1594	0.9990	*rps*2	1	0.0583	1.5804
*psa*A	1	**0.0000**	5.5339	*rps*3	1	**0.0019**	2.9447
*psa*B	1	**0.0000**	5.3084	*rps*4	1	**0.0062**	2.5373
*psa*C	1	0.1711	0.9537	*rps*7	**0.0130**	1	2.2541
*psa*I	**0.0482**	1	1.6756	*rps*8	1	0.3590	0.3619
*psa*J	1	0.4104	0.2270	*rps*11	1	0.0638	1.5339
*psa*M	0.4967	1	0.0084	*rps*12	1	0.1016	1.2795
*psb*A	1	**0.0004**	3.4212	*rps*14	1	0.0984	1.2977
*psb*B	1	**0.0003**	3.5747	*rps*15	1	**0.0070**	2.4949
*psb*C	1	**0.0002**	3.6848	*rps*18	1	0.1515	1.0343
*psb*D	1	**0.0045**	2.6582	*rps*19	1	0.0863	1.3722
*psb*E	1	0.0642	1.5310	*ycf*1	**0.0000**	1	4.0848
*psb*F	0.0587	1	1.5769	*ycf*2	**0.0156**	1	2.1793
*psb*H	**0.0124**	1	2.2732	*ycf*3	1	0.0813	1.4051
*psb*I	1	0.1810	0.9151	*ycf*4	1	0.0531	1.6274
*psb*J	0.0916	1	1.3389				

Results shown are overall average of all ingroup pairwise comparisons, with significance at *P *≤ 0.05 indicated in **bold**.

### Phylogenetic Resolution in Non-Random and Randomized Data Partitions

**Figure 2:**
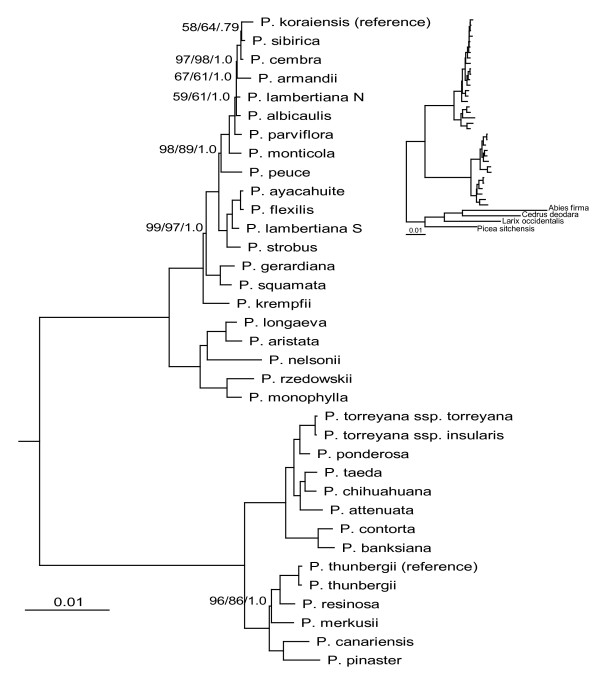
**Phylogenetic relationships of 35 pines and four outgroups as determined from full plastome sequences**. Support values are only shown for nodes with bootstrap/posterior probability values less than 100%/1.0, and are shown as ML bootstrap/MP bootstrap/BI posterior probability. Branch lengths calculated through RAxML analysis, and correspond to scale bar (in units of changes/nucleotide position). Inset shows topology of outgroups relative to ingroup accessions.

**Figure 3:**
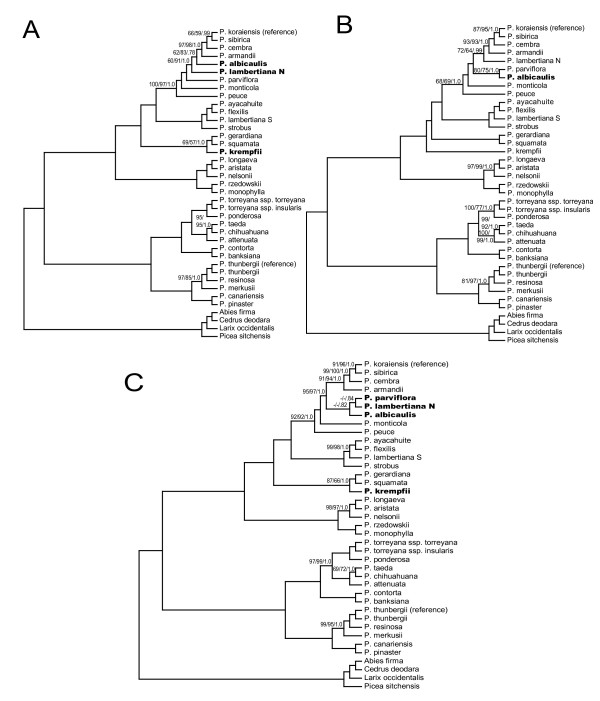
**Phylogenetic relationships of 35 pines and four outgroups as determined from different data partitions**. **A) **Full alignment without *ycf*1 and *ycf*2. **B) **Exon nucleotide sequences. **C) **Exon nucleotide sequences without *ycf*1 and *ycf*2. Support values are only shown for nodes with bootstrap/posterior probability values less than 100%/1.0, and are shown as ML bootstrap/MP bootstrap/BI posterior probability. Dashes indicate < 50% bootstrap support or < .50 posterior probability. Accessions whose position differs from that in full alignment analysis indicated in bold.

**Figure 4:**
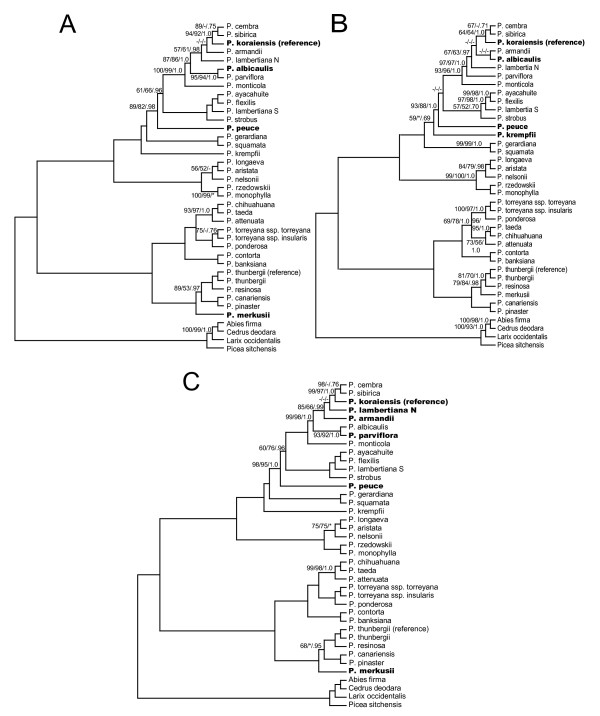
**Phylogenetic relationships of 35 pines and four outgroups as determined from *ycf1 *and *ycf2 *partitions**. **A) ***ycf*1 only. **B) ***ycf*2 only. **C) ***ycf*1 and *ycf*2 combined. Support values are only shown for nodes with bootstrap/posterior probability values less than 100%/1.0, and are shown as ML bootstrap/MP bootstrap/BI posterior probability. Dashes indicate < 50% bootstrap support or < .50 posterior probability, * indicates topological difference between either parsimony or Bayesian analyses and ML. Accessions whose position differs from that in full alignment analysis indicated in **bold**.

Full alignment partitions yielded a higher proportion of highly supported nodes, with 88 to 91% (29 to 30/33) of ingroup nodes resolved with bootstrap support ≥ 95% in likelihood analysis. The four largest data partitions tested (full alignment and concatenated exon nucleotides, both with and without *ycf*1 and *ycf*2) yielded results that were topologically identical with the exception of four taxa (*P. albicaulis*, *P. krempfii*, *P. lambertiana *N, *P. parviflora*) (Figures 2 and 3). In addition, support for the branching order of *P. cembra*, *P. koraiensis *and *P. sibirica *was low in full alignment partitions. Topological differences were found to be significant according to Shimodaira-Hasegawa comparisons of the full alignment topology to two of the other major partitions (full alignment and exon nucleotides without *ycf*1 and *ycf*2). Trends in significance were most strongly influenced by the two alternative positions of *P. krempfii *(Figure 2 vs. Figure 3A, C; Table 4). With the exception of *P. krempfii*, areas of topological uncertainty reside in a single clade that historically has lacked internal resolution (subsection *Strobus*) [20-22]. Coalescent estimations suggest that these poorly resolved subsection *Strobus *haplotypes diverged in rapid succession relative to the age of their shared nodes (0.009 to 0.44 coalescent units, or ca. 90,000 to 450,000 years) (Table 5). A putative chloroplast capture event in *P. lambertiana *previously documented [5] was also supported with whole-plastome results. Substantial resolution was achieved in analyses of *ycf*1 and *ycf*2 data partitions, however we observed several topological differences from the full alignment with high support (primarily involving the species discussed above) (Figure 4).

**Table 4:** Shimodaira-Hasegawa test results.

*P. krempfii *topologies	*P. albicaulis*, *P. lambertiana *N, *P. parviflora *topologies	*P*-value
**Figure 2 vs. 3A**	**2 vs. 3A**	0.011*
Figure 2 vs. 2	**2 vs. 3A**	0.153
**Figure 2 vs. 3A**	2 vs. 2	0.024*

Figure 2 vs. 3B	**2 vs. 3B**	0.351
**Figure 2 vs. 3A**	**2 vs. 3B**	0.063
**Figure 2 vs. 3A**	2 vs. 2	0.063

**Figure 2 vs. 3C**	**2 vs. 3C**	0.005*
Figure 2 vs. 2	**2 vs. 3C**	0.050
**Figure 2 vs. 3C**	2 vs. 2	0.024*

Results of significance testing for topology comparisons of the full alignment (Figure 2) versus the three other largest data partitions (Figure 3). For each set of comparisons, the first row represents comparison of unmodified maximum likelihood topologies. In the second and third rows the positions of *P. krempfii *and *P. albicaulis *- *P. lambertiana *N - *P. parviflora *were modified as indicated. Topologies that differ within a comparison are indicated in **bold**. Significant topological differences at *P *< 0.05 are indicated with an asterisk.

**Table 5:** Estimated divergence times of poorly resolved nodes

Node	ML branch length (substitutions/site)	Estimated divergence time
*P. krempfii - *section *Quinquefoliae*	0.000370	1126539 22531 0.113/1.13
*P. parviflora *- *P. albicaulis*	0.000144	442057 8841 0.044/0.44
*P. albicaulis *- *P. lambertiana *N	0.000030	92095 1842 0.009/0.09
*P. cembra *- *P. koraiensis*/*sibirica*	0.000085	260936 5219 0.026/0.26

All divergence time estimates assume a chloroplast mutation rate of 3.26 × 10^-10 ^substitutions/site/year. Coalescent units reported are based on either high (100,000) or low (10,000) effective population (N_e_) sizes. Maximum likelihood (ML) branch lengths are shown as substitutions/site. Estimated divergence times are presented in years (top), generations (middle) and coalescent units for high/low N_e _(bottom).

**Figure 5:**
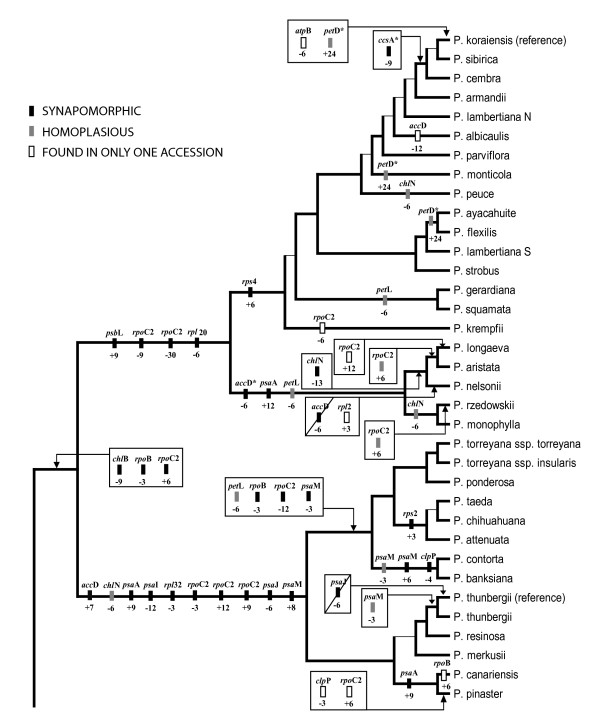
**Phylogenetic distribution of exon coding indel mutations in sampled *Pinus *accessions**. Exon names given above boxes, size of indel (bp) and polarity ("+" = insertion, "-" = deletion) given below boxes. Polarity of events determined by comparison to most distant outgroups. Due to the apparent high rate of indel formation in *ycf*1 and *ycf*2, these loci were not able to be confidently scored for indels and are not included in this diagram. Events for only the first copy of *psa*M are reported. Branching order of tree corresponds to RAxML analysis of complete alignment. Diagonal lines represent putative reversals of indel events. * indicates missing data for one or more accessions of clade. Thin internal branches correspond to ML bootstrap support < 95% or topological difference in four largest data partitions (full alignment and exon nucleotides, with and without *ycf*1 and *ycf*2).

**Figure 6:**
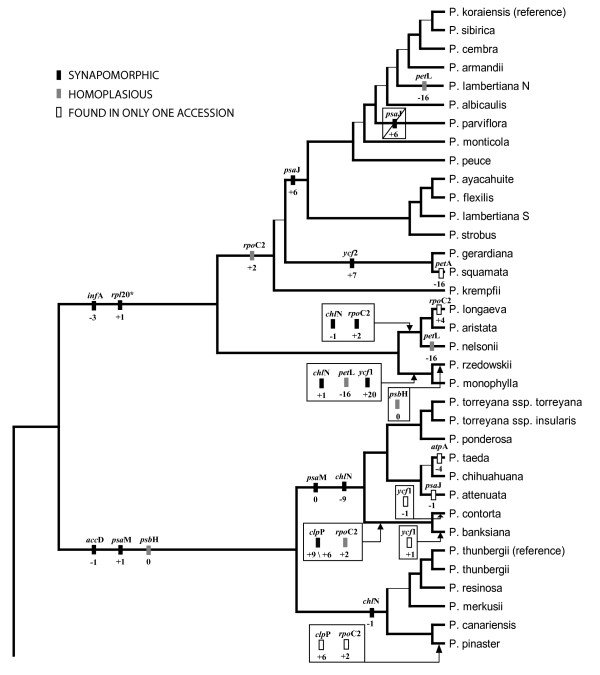
**Phylogenetic distribution of stop codon mutations in sampled *Pinus *accessions**. Exon names given above boxes, amino acid shift relative to stop codon position in outgroups given below boxes. Polarity of events determined by comparison to most distant outgroups; "+" signifies extension of coding region due to stop codon mutation, "-" signifies shortening. The value of zero for the *psb*H- and *psa*M-associated events corresponds to events that alter the original stop codon without altering the total number of codons in the locus. Events for only the first copy of *psa*M are reported. Diagonal line represents a putative reversal in *psa*J of *P. parviflora*. Branching order of tree corresponds to RAxML analysis of complete alignment. * indicates missing data for one or more accessions of clade. Thin internal branches correspond to ML bootstrap support < 95% or topological difference in four largest data partitions (full alignment and exon nucleotides, with and without *ycf*1 and *ycf*2).

Of the 71 exon coding indels and stop codon shifts identified, 35 mapped unambiguously to monophyletic groups (that is, no accessions in a group were missing data for that event) (Figures 5 and 6). All of these groups had strong support in nucleotide-based phylogenetic analyses (100% likelihood and parsimony bootstrap support). The remainder of these events were primarily either putatively monophyletic (missing data in one or more members of a clade) or showed strong evidence of homoplasy (Figures 5 and 6).

**Figure 7:**
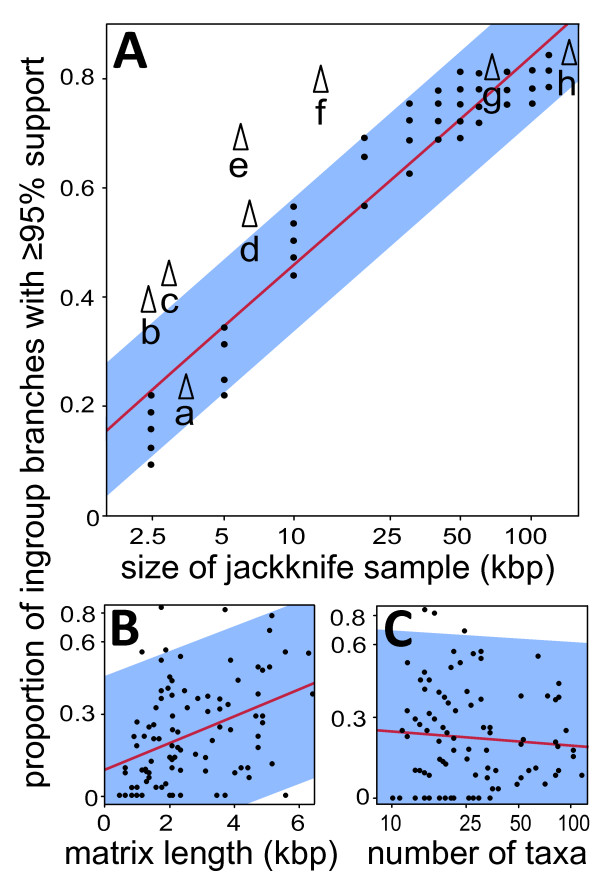
**Relationships between matrix size and resolution in current study and meta-analysis of published studies**. **A) **Parsimony resolution of jackknifed partitions (black circle) of full alignment of current study. Labelled data points (triangle) represent resolution of the following: a - Wang et al. [22], b - Gernandt et al. [21], c - Eckert and Hall [20], d - *ycf*2, e - *ycf*1, f - combined *ycf*1 and *ycf*2, g - exon nucleotides, h - complete alignment. **B) **Relationship between matrix length and phylogenetic resolution in published studies (N = 99). **C) **Relationship between number of taxa and phylogenetic resolution in published studies (N = 99). Regression lines are shown in red; 95% confidence intervals shown in blue. X-axes of A, B and C and Y-axes of B and C are in log scale.

In parsimony analyses of variable-sized jackknife samples of our full alignment, nodal support showed a strong positive correlation with the length of the nucleotide matrix (proportion nodes ≥ 95% = -1.0808 + 0.38497*log_10 _[matrix size, bp]; r^2 ^= 0.915, *P *< 0.0001) (Figure 7A). Resolution of full alignment and exon nucleotide partitions was indistinguishable from random jackknife samples of comparable size, indicating similar phylogenetic content of these partitions and corresponding similar-sized random genomic subsamples. Partitions consisting of *ycf*1 and *ycf*2 - in particular *ycf1*, and *ycf1 *and *ycf2 *combined - showed significantly higher resolution than the genome-wide average (Figure 7A). The concatenated partition *ycf1 *+ *ycf2 *(13.1 kb; 77.4% nodes ≥ 95% bootstrap support) yielded only slightly less phylogenetic resolution than all exons combined (62.3 kb; 80.6% nodes ≥ 95% bootstrap support) in parsimony analysis.

### Comparisons to Previous *Pinus *Phylogenies

Previous cpDNA based estimates of infrageneric relationships in *Pinus *[20-22] sampled the same species and/or lineages as our study, and inferred relationships using 2.82 to 3.57 kb of chloroplast DNA. Results of these studies are largely consistent with our results, although highly supported nodes (≥ 95%) accounted for only 13 to 23% of the total ingroup nodes (23% to 42% if [20,21] adjusted to match our species composition). The empirical results of these studies fell within or close to the 95% prediction intervals established from our jackknife resampling response from our full genome alignment (Figure 7A), indicating that the loci used in prior studies (primarily *rbc*L and *mat*K) are similarly informative as a comparable sample of random nucleotides from the chloroplast genome.

### Meta-Analysis of Published Infrageneric Studies

From our sampling, infrageneric analyses in plants published from 2006 to 2008 were typically based on 2574 aligned bp (95% bootstrap confidence interval: 2,292, 2,864) of sequence data, evaluated 31.7 ingroup species (95% bootstrap confidence interval: 20.2, 43.2), and resolved 22.6% of nodes at ≥ 95% bootstrap support (95% bootstrap confidence interval: 18.6, 26.5). Regression analysis shows that the proportion of highly resolved nodes in these studies is significantly and positively correlated with matrix length (F_1,96 _= 18.032; r^2 ^= 0.149; *P *< 0.0001) but not the number of included taxa (F_1,97 _= 0.546; *r*^2 ^= 0.006; *P *= 0.461), although there was a negative trend in the latter (Figure 7B, C). Our current sample size is typical in the number of taxa sampled, but both matrix length (132.7 kb) and the proportion of highly bootstrap-supported nodes (84.8% parsimony, 90.3% maximum likelihood) were substantially higher.

## Discussion

**Figure 8:**
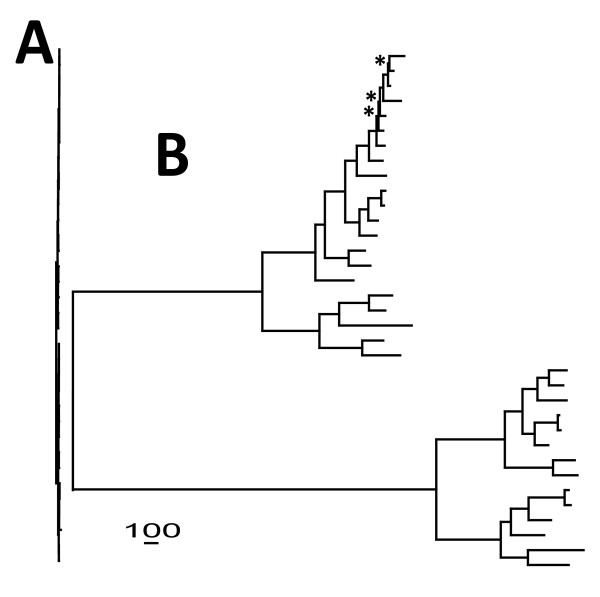
**Comparative phylogenetic resolution of *Pinus *species used in this study**. Resolution from **A) **two chloroplast loci [21] and **B) **our complete alignment. Distance bar corresponds to 100 nucleotide changes, and is scaled for either tree. * indicate branches with < 95% (likelihood) bootstrap support in B) (likelihood and parsimony topologies were completely congruent).

Our results highlight that whole plastome sequencing is now a feasible and effective option for inferring phylogenies at low taxonomic levels. Compared to previous chloroplast-based phylogenetic analyses in *Pinus*, our data matrix contained approximately 60 times more phylogenetically informative characters resulting in an approximately two- to four-fold increase in the proportion of highly resolved nodes (after adjusting results of previous studies to match our species composition) (Figure 8, Table 2). An important question arising from these comparisons is whether the difference in resolution is entirely attributable to the increase in nucleotides, or whether the genomic partitions sequenced in prior studies were less informative on average than the rest of the genome. In fact, the resolution provided by loci used in previous *Pinus *studies is indistinguishable from or slightly greater than that of comparably sized random genomic subsamples from our full alignment. Combined with the strong correlation between resolution and the size of random genomic subsample, this suggests that the increase in resolution in this study is primarily due to the increase in matrix length. This is further supported by a significant relationship between resolution and matrix length in a broad sampling of chloroplast-based infrageneric phylogenies. Based on these results, we predict that whole-plastome analysis will yield similar gains in phylogenetic resolution not only in the genus *Pinus *but for most land plant genera. On the other hand, it is apparent that even the entire chloroplast genome may be insufficient to fully resolve the most rapidly radiating lineages. In this regard, our results are reflective of previous analyses of ancient rapid radiations wherein nodal resolution does not scale proportionately to the length of sequence analyzed [27,28]. Notably, the position of *P. krempfii *was significantly different between the four largest data partitions (Table 4), even though this species does not appear to be associated with a rapid radiation (Table 5). This result is not completely unexpected, as this species has previously been difficult to place phylogenetically [29,30]. An unequivocal resolution of this species will likely require the inclusion of multiple nuclear loci [30].

When considering recent divergence, the disproportionately high mutation rate in *ycf*1 (and *ycf*2, to a lesser extent) demonstrated here is of importance, and mirrors findings in other plant taxa [31,32] and recently in *Pinus *subsection *Ponderosae *[33]. These loci should be informative for phylogenetic studies in recently-diverged clades or in population-level studies in a range of plant species. Discretion is advised, however, as *ycf*1 (and possibly *ycf*2) appears to be a target of positive selection at least in *Pinus *and may reflect adaptive episodes rather than neutral genealogies. In likelihood analyses of *ycf*1 and *ycf*2, we observed several topological differences from the full alignment at the subsectional level, further demonstrating that caution must be taken in drawing phylogenetic conclusions from these two loci. Although we were able to confidently score small structural changes (indels and stop codon shifts) for all other exons, it was not possible to score indels for *ycf*1 and *ycf*2 due to the apparent high rate of indel formation in these loci. In all other loci examined, small structural changes only delineated clades with concurrent high support from nucleotide-based analyses (both in present study and [20-22]), and thus are likely to be of limited use in species or population level discrimination. It is not clear whether this will also be the case in *ycf*1 and *ycf*2.

It is reasonable to ask whether increased resolution is worth the effort of assembling whole plastomes. Considering the conservative nature of bootstrap measures [34-37], systematists often accept bootstrap values of ≥ 70% as reliable indicators of accurate topology [36]. Simulation studies [34], however, have demonstrated greatly increased accuracy (approximately 42×) with bootstrap values ≥ 95% versus ≥ 70%, and the initial formulation of the phylogenetic bootstrap used ≥ 95% as the threshold for topological significance [38]. Our results similarly support using a 95% bootstrap support cutoff for conclusive evidence as in both areas of topological differences, more than one clade received bootstrap support ≥ 70% by analysis of alternate data partitions. It is probable that conflicting topologies with ≥ 70% but < 95% bootstrap support accurately reflect data partitions yet may not represent the plastome phylogeny, and here the use of entire organelle genomes makes it possible to adopt more conservative criteria of nodal support. There are further biological reasons why an organellar phylogeny (essentially a single-gene estimate) may not accurately represent the organismal phylogeny; these include interspecific hybridization, incomplete lineage sorting, and stochastic properties of the coalescent process. Nonetheless, phylogenetic reconstruction based on complete organellar sequences may facilitate the detection of such phenomena, by reducing errors and uncertainty due to insufficient sampling of DNA sequence.

## Conclusion

Plastome sequencing is now a reasonable option for increasing resolution in phylogenetic studies at low taxonomic levels and will continue to become an increasingly simple process. As sequencers evolve to even higher capacity and multiplexing becomes routine in the near future, this will allow more extensive taxon and genomic sampling in phylogenetic studies at all taxonomic levels. It is estimated that sequencing capacity on next generation platforms will approach 100 gigabase pairs per sequencing run by the end of 2009. For perspective, this is sufficient sequence capacity to produce all 100 genus-level data sets used in our meta-analysis (including ours) at greater than 100× coverage depth in a single sequencing run. Based on the estimates of Cronn et al. [9], this sequencing capacity would also allow the simultaneous sequencing of several thousands of animal mitochondria, which could greatly benefit low-level taxonomic or population-based studies in animals that currently tend to rely on relatively short sequences from many individuals [39]. It is also clear that these improvements could enable other pursuits that are currently hindered by limited sequencing capacity, such as identification of plants by diagnostic DNA sequences (DNA barcoding). The recently agreed upon two locus chloroplast barcode for plants claims only 72% unique identification to species level[40]. Based on results herein, whole plastome sequences have the potential to be more highly discriminating and efficient plant DNA barcodes; in fact, the possibility of plastome- and mitome-scale barcodes has been raised previously [41]. Results in this area (as well as in phylogenetic and phylogeographic analyses) will be impacted particularly if advances in target isolation and enrichment [13-15] and streamlining sample preparation [17] prove globally effective.

## Methods

### DNA Extraction, Amplification and Sequencing

DNA extraction, amplification and sequencing are described in and followed Cronn et al. [9], with 4 bp multiplex tags, replacing the original 3 bp tags (Table 1). For one sample, *P. ponderosa*, additional reads from three non-multiplexed lanes of genomic DNA were also included.

### Sequence Assembly and Genome Alignments

Sequence assembly and alignment are described in and followed Whittall et al. [42]. An analysis of interspecific recombination was conducted using RDP(Recombination Detection Program) v. 3.27 [43]. Rather than using the full genomic alignment, which was too memory-intensive, concatenated nucleotide sequences for 71 exons common to all accessions were used (reflective of order on the plastome). Subgenera were investigated separately as members of opposing subgenera appear incapable of hybridization [44]. Each subgenus was checked for recombination events using standard settings for several recombination-detection strategies, including: RDP [45], GeneConv [46], Chimaera [47], MaxChi [48], BootScan [49], and SiScan [50]. A total of 24 putative recombination events were identified. On close investigation, all events involved one or more of the following: misalignment, autapomorphic noise coupled with missing data, and amplification of pseudogenes. In cases of misalignment, alignments were corrected prior to subsequent phylogenetic analyses. In cases of amplification of pseudogenes, the entire amplicon for the accession involved was turned to Ns. Inspection of the alignment also revealed that some amplicons in some accessions had failed to amplify, or amplified apparently paralogous loci (evidenced by substantially higher divergence). These regions were masked in affected accessions. The locus *mat*K was determined to be a putative paralog in several accessions, and in four (*P. armandii*, *P. lambertiana *S, *P. albicaulis*, and *P. ayacahuite*) it was replaced with Sanger sequence [5]. We also replaced 2180 bp of poor quality sequence of the locus *ycf*1 in *P. ponderosa *with Sanger sequence. In all accessions amplified by PCR, the regions adjacent to primer sites typically had low coverage, while primers had very high coverage, thus primer-flanking regions (where problematic) and the primers were also excluded. It was also determined through Sanger sequencing that a 600 bp region of the previously published *P. koraiensis *plastome (positions 48808 to 49634 in GenBank AY228468) is apparently erroneous. This region was removed and reference guided analysis was rerun for this amplicon.

Aligned sequences were annotated using DOGMA (Dual Organellar Genome Annotator) [51] with manual adjustments to match gene predictions from GenBank and the Chloroplast Genome Database http://chloroplast.cbio.psu.edu/. Exons were evaluated for reading frame and translations, and validity of exon mutations was judged based on presence in de novo sequence, effect on the resulting polypeptide sequence, and sequence coverage depth.

### Phylogenetic Analyses

Sequence data was analyzed using all genome positions and concatenated nucleotide sequence from 71 exons common to all pine accessions; both partitions were analyzed with and without the loci *ycf*1 and *ycf*2. A relatively short (approximately 630 bp) repetitive stretch of the locus *ycf*1 of subgenus *Strobus *accessions was masked in all analyses due to alignment ambiguity. The loci *ycf*1 and *ycf*2 (ca. 14 kb combined) were also analyzed individually and together.

Maximum Likelihood (ML) phylogenetic analyses were performed through the Cipres Web Portal http://www.phylo.org/portal/Home.do using RAxML bootstrapping with the general model of nucleotide evolution (GTR+G) [52] and automatically determined numbers of bootstrap replicates. Bayesian inference analyses (BI) were performed using MrBayes v. 3.1.2 [53] using the GTR+G+I model, which was selected using MrModelTest v. 2.3 [54] under both Aikake Information Criterion and Hierarchical Likelihood Ratio Test frameworks. Each analysis consisted of two runs with four chains each (three hot and one cold chain), run for 1000000 generations with trees sampled every 100 generations. The first 25% percent of trees from all runs were discarded as burn-in. Unweighted maximum parsimony analyses (MP) of data partitions were conducted in PAUP* (Phylogenetic Analysis Using Parsimony (*and other methods)) v. 4.0b10 [55] by heuristic search with 10 replicates of random sequence addition, tree bisection and reconnection branch swapping and a maxtrees limit of 1,000. Non-parametric bootstrap analysis was conducted under the same conditions for 1,000 replicates to determine branch support.

Topological differences between the full alignment topology and each of the three other largest data partitions (full alignment without *ycf*1 and *ycf*2, and exon nucleotides both with and without *ycf*1 and *ycf*2) were tested for significance using the Shimodaira-Hasegawa test [56] with resampling estimated log-likelihood (RELL) bootstrapping (1,000 replicates) under the GTR+G model of evolution. To further determine which topological differences were most influential, tests were repeated with the positions of topology-variable accessions alternately modified to match the full alignment topology. In total, the full alignment data set was compared to nine different topologies.

Exon indels and stop codon shifts were mapped onto the topology determined by ML analysis of the full alignment by parsimony mapping using Mesquite v. 2.6 (Maddison and Maddison, http://mesquiteproject.org). Tests of selection for exons were performed in MEGA v. 4.0 [57] using the codon-based Z-test for selection, with pairwise deletion and the Nei-Gojobori (*P*-distance) model; variance of the differences were computed using the bootstrap method with 500 replicates.

### Estimation of Divergence Times for Poorly Resolved Nodes

Divergence times for four nodes with topological uncertainty (*P. albicaulis *- *P. lambertiana *N - *P. parviflora*, *P. sibirica *- *P. cembra *- *P. koraiensis*, *P. krempfii*-section *Quinquefoliae *of subgenus *Strobus*) were estimated according to Pollard et al. [58]. Chloroplast mutation rate was estimated by averaging maximum and minimum mutation rates for Pinaceae chloroplast genomes from two previous studies [59,60] and assuming a generation time of 50 years [61]. Two estimates were calculated for each node using either low (10,000) or high (100,000) effective population size [23].

### Effect of Character Number on Phylogenetic Resolution

#### Empirical data from Pinus genomes

Variable-size random subsamples of the full alignment were tested under the parsimony criteria using PAUP* v. 4.0b10 (the faststep option was used for all but the two smallest partitions due to time considerations). Eleven partition sizes were tested (2.5, 5, 10, 20, 30, 40, 50, 60, 80, 100 and 120 kb) in five replicates each, with resolution measured as the percentage of ingroup nodes produced with ≥ 95% jackknife support. Relationships between partition size and ingroup resolution were estimated using least squares regressions, and 95% confidence limits for individual points were estimated based on linear regression using SAS JMP 7.0.1 (S.A.S. Institute, Inc., http://www.jmp.com/). Our full alignment, exon nucleotides and *ycf*1/*ycf*2 partitions were analyzed under the same parsimony criteria for comparison, as were the alignments of [20-22]. Accessions from Gernandt et al. and Eckert et al. [20,21] were pruned to include only taxa common to our sampling; the original analysis of Wang et al. [22] was used since this data matrix was not available for alternative phylogenetic analyses.

#### Meta-Analysis of Published Studies

We evaluated 99 phylogenetic analyses from 86 studies published between 2006 and 2008 in Systematic Botany, Systematic Biology, American Journal of Botany, Taxon, Molecular Phylogenetics and Evolution, and Annals of the Missouri Botanical Garden [see additional file 2]. Analyses were selected based on: 1) the presented phylogeny was based solely on chloroplast DNA sequence; 2) the analysis included ≥ 10 species from a monophyletic genus; 3) there were more inter- than intra-specific taxa analyzed within the genus; 4) parsimony-based bootstrap or jackknife values were presented. Ingroup branches with bootstrap support ≥ 95%, the number of ingroup taxa and the aligned base pairs used in the analysis were recorded for each case. The authors' taxonomic interpretations were accepted in instances of taxonomic uncertainty. Conspecific clades were treated as one taxon unless clearly differentiated from one another, and internal bootstrap values were disregarded. The number of branches with bootstrap support ≥ 95% was regressed both on the number of aligned base pairs and the number of taxa (both log-transformed to meet assumptions of normality and equal variances).

### Data Deposition

Illumina sequencing reads and quality scores have been deposited in the NCBI SRA database as accession SRA009802. New sequences have been deposited in GenBank as accessions FJ899555-FJ899583.

### Accession numbers cited in manuscript

[GenBank FJ899555-FJ899583, EU998739-EU998746, SRA009802]

## Abbreviations

BI: Bayesian Inference; bp: base pairs; cpDNA: chloroplast DNA; kb: kilobase; ML: maximum likelihood; MP: maximum parsimony; MPS: massively parallel sequencing.

## Authors' contributions

MP obtained and assembled the plastome data, conducted the phylogenetic and statistical analyses, conducted the meta-analysis and drafted the manuscript. RC and AL conceived the study and contributed to data collection, data analysis and manuscript writing. All authors read and approved the final manuscript.

## Supplementary Material

Additional file 1 **Coverage Densities**. A) Subgenus *Strobus*. B) Subgenus *Pinus*. C) Outgroups. Horizontal bars in charts indicate median coverage level for an amplicon. Click here for file

Additional file 2 **Meta-Analysis Details**. Details of studies included in meta-analysis of bootstrap distributions. Click here for file
