# Supplementary figures and images for: Increasing phylogenetic resolution at low taxonomic levels using massively parallel sequencing of chloroplast genomes
Source: BMC Biol. 2009 Dec 2;7:84. doi: 10.1186/1741-7007-7-84 (PMC2793254; doi:10.1186/1741-7007-7-84)

A

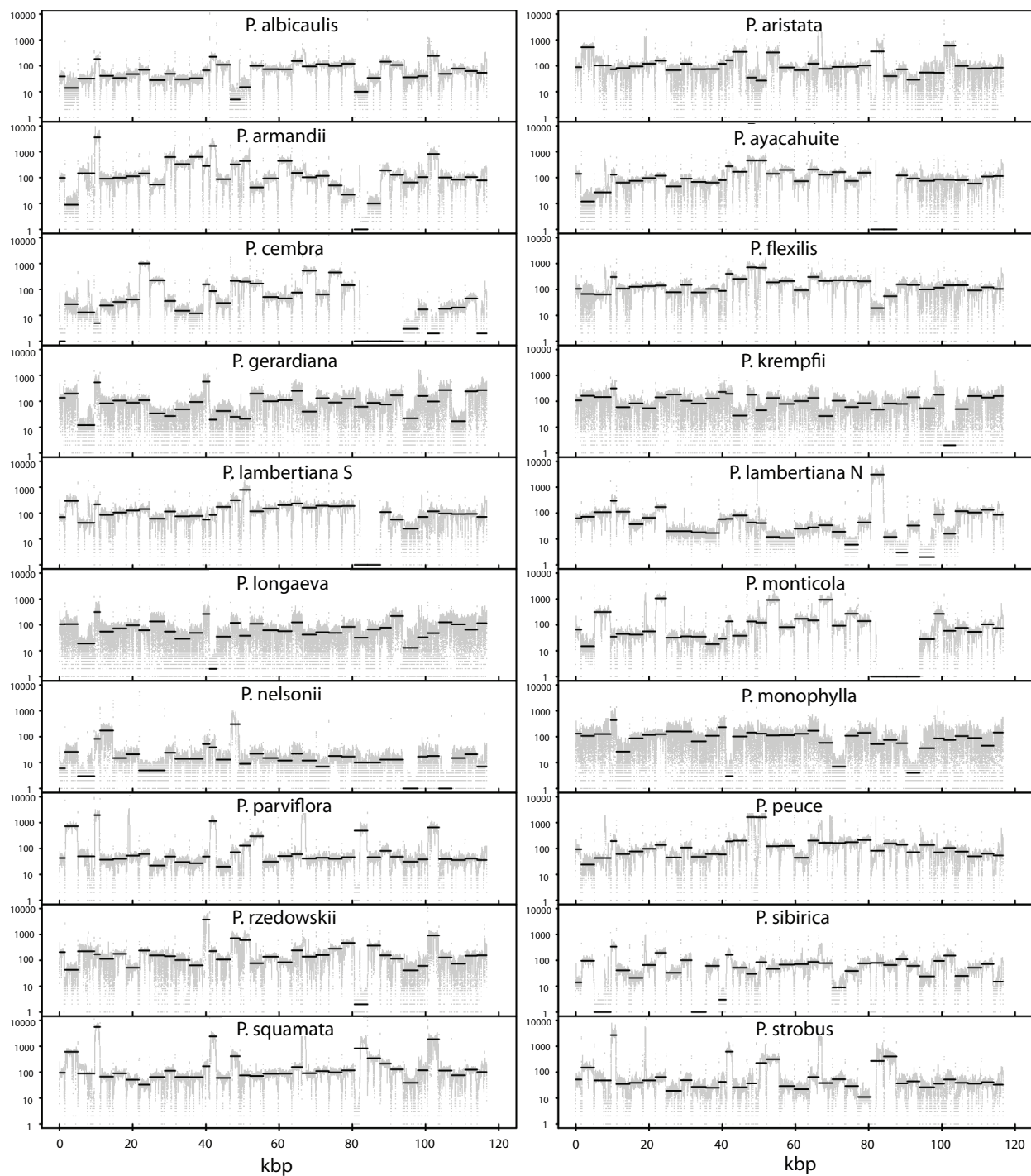

**B**

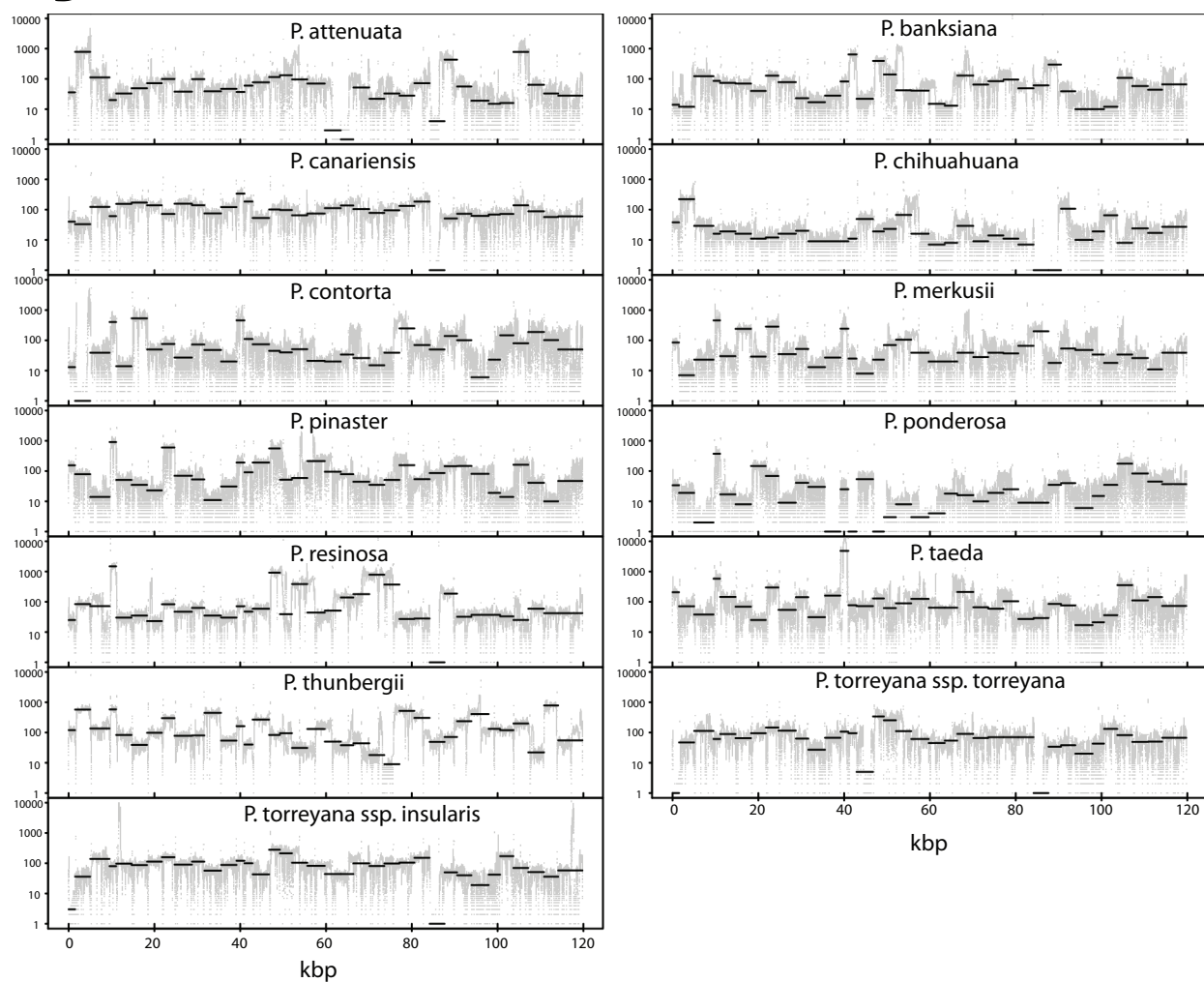

**C**

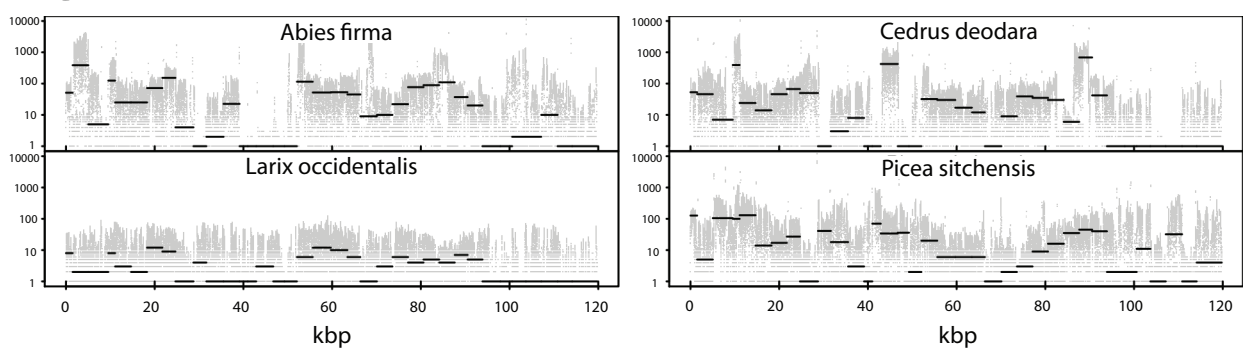

Supplement: Additional file 1 — Coverage Densities. A) Subgenus Strobus. B) Subgenus Pinus. C) Outgroups. Horizontal bars in charts indicate median coverage level for an amplicon. [file 1741-7007-7-84-S1.PDF]
